# The predictive value of TIMP-2 and IGFBP7 for kidney failure and 30-day mortality after elective cardiac surgery

**DOI:** 10.1038/s41598-020-80196-2

**Published:** 2021-01-13

**Authors:** Kevin Esmeijer, Abraham Schoe, L. Renee Ruhaak, Ellen K. Hoogeveen, Darius Soonawala, Fred P. H. T. M. Romijn, Maryam R. Shirzada, Jaap T. van Dissel, Christa M. Cobbaert, Johan W. de Fijter

**Affiliations:** 1 Department of Nephrology, Leiden University Medical Center (Building 1, C7-Q), Albinusdreef 2, 2333 ZA Leiden, The Netherlands; 2 Department of Clinical Epidemiology, Leiden University Medical Center, Leiden, The Netherlands; 3 Department of Intensive Care, Leiden University Medical Center, Leiden, The Netherlands; 4 Department of Clinical Chemistry and Laboratory Medicine, Leiden University Medical Center, Leiden, The Netherlands; 5 Department of Internal Medicine, Haga Teaching Hospital, Den Haag, The Netherlands

**Keywords:** Acute kidney injury, Continuous renal replacement therapy, Interventional cardiology

## Abstract

Acute kidney injury (AKI) is an important risk factor for chronic kidney disease, renal replacement therapy (RRT), and mortality. However, predicting AKI with currently available markers remains problematic. We assessed the predictive value of urinary tissue inhibitor of metalloprotease-2 (TIMP-2) and insulin-like growth factor-binding protein 7 (IGFBP7) regarding the need for RRT, and 30-day mortality, in elective cardiac surgery patients. In 344 elective cardiac surgery patients, we measured urinary TIMP-2 and IGFBP7 and serum creatinine at baseline and directly after surgery. Discrimination of both urinary biomarkers was assessed by the C-statistic. Model improvement for each biomarker when added to a basic model containing serum creatinine and duration of surgery was tested by the net-reclassification index (cf-NRI) and integrated discrimination index (IDI). At baseline, mean age was 66 years and 67% were men. Of all patients, 22 required RRT following surgery. IGFBP7 pre- and post-surgery and change in TIMP-2 during surgery predicted RRT with a C-statistic of about 0.80. However, a simple model including baseline serum creatinine and duration of surgery had a C-statistic of 0.92, which was improved to 0.93 upon addition of post-surgery TIMP-2 or IGFBP7, with statistically significant cf-NRIs but non-significant IDIs. Post-surgery TIMP-2 and IGFBP predicted 30-day mortality, with C-statistics of 0.74 and 0.80. In conclusion, in elective cardiac surgery patients, pre- and peri-operative clinical variables were highly discriminating about which patients required RRT after surgery. Nonetheless, in elective cardiac surgery patients, urinary TIMP-2 and IGFBP7 improved prediction of RRT and 30-day mortality post-surgery.

## Introduction

Acute kidney injury (AKI) is an important risk factor for chronic kidney disease, need of renal replacement therapy (RRT) and mortality^1,2^. AKI is frequently caused by medical interventions and their side effects, such as treatment with nephrotoxic medication or peri-operative hypotension^3^. In particular, patients undergoing cardiac surgery are at high risk of AKI. The diagnosis of AKI is based on a rise in serum creatinine and/or reduction of urinary output, according to the KDIGO criteria^4^. However, usefulness of both parameters is limited in the early stages of AKI and to identify patients at risk for AKI^5^. As a consequence, AKI is often diagnosed after irreversible renal damage has already occurred. Alternative markers, which have the potential to identify patients at high risk of AKI before cardiac surgery or start of nephrotoxic medication to escalate preventive measures, are thus needed.

Recently, two urinary cell-cycle arrest markers, tissue inhibitor of metalloprotease-2 (TIMP-2) and insulin-like growth factor-binding protein 7 (IGFBP7), were approved by the U.S. Food and Drug Administration for clinical AKI prediction. Urinary levels of TIMP-2 and IGFBP7 increase upon acute kidney damage, owing to changes in tubular filtration, reduction of reabsorption, and leakage due to tubular damage^6^. TIMP-2 is preferentially secreted by distal tubule cells, while IGFBP7 is mainly secreted by proximal tubule cells^7^. The differential secretion localization of both biomarkers may characterize the extent and mechanism of kidney damage. Multiple studies showed good predictive performance of both biomarkers for AKI, in heterogeneous intensive care populations^8^.

The value of both biomarkers to predict AKI, need for RRT or death in elective cardiac surgery patients, is unclear. Therefore, we aimed to validate the predictive value of IGFBP7 and TIMP-2 regarding risk of severe AKI needing RRT. Since occurrence of AKI also increases mortality risk and may lead to longer hospital admissions, we investigated as secondary outcomes the predictive value of both biomarkers with respect to length of intensive care unit (ICU) stay and 30-day mortality.

## Results

### Baseline characteristics

Baseline characteristics are presented in Table 1 for all patients, and separately for patients with or without need of RRT. The majority (n = 16/22) of all patients who required RRT started within 3 days post-surgery. For all patients, mean age was 66 years, 67% were men and the proportion of patients with cardiovascular comorbidity was high (Table 1). Eighteen patients died within 30 days. Patients requiring RRT compared with patients not needing RRT, had lower baseline estimated glomerular filtration rate (eGFR), higher APACHE IV score, longer duration of surgery, and had more frequently hypertension and heart failure. Baseline biomarker levels did not correlate with serum creatinine (*p* > 0.3); only post-surgery TIMP-2 levels weakly correlated with serum creatinine (Pearson correlation 0.13, *p* = 0.04).

**Table 1:** Baseline characteristics of all 344 elective cardiac surgery patients and stratified according to start of renal replacement therapy (RRT).

	All patients (n = 344)	No RRT (n = 322)	RRT (n = 22)
Age, y	66 ± 11	66 ± 11	68 ± 14
Sex, % men	65	64	73
Current smoker %	23	23	29
Body-mass index, kg/m^2^	27 ± 4	27 ± 4	27 ± 6
Duration of surgery, min	292 ± 121	289 ± 117	402 ± 202
CPB-time, min	155 ± 72	152 ± 69	241 ± 147
APACHE IV score^a^	50 ± 16	49 ± 15	79 ± 39
EuroSCORE^b^	5.6 ± 2.8	5.5 ± 2.7	7.3 ± 4.6
Hypertension,^c^ %	47	47	36
BP-lowering drugs,^d^ %	62	62	77
ACE-inhibiting drugs, %	58	57	68
Lipid-modifying drugs,^d^ %	61	61	59
Diabetes,^e^ %	21	21	23
Heart failure, %	17	16	50
Serum cystatin C, mg/L	1.04 ± 0.31	1.04 ± 0.29	1.79 ± 1.28
Serum creatinine,^f^ µmol/L	82 ± 26	81 ± 22	140 ± 99
eGFR_cysC_, mL/min/1.73 m^2^	76 ± 23	76 ± 23	49 ± 57
eGFR_cr-cysC_, mL/min/1.73 m^2^	79 ± 21	79 ± 20	51 ± 52
Urinary protein, g/L	0.15 ± 0.23	0.15 ± 0.22	0.22 ± 0.55
Urinary creatinine, mmol/L	10.9 ± 6.1	10.9 ± 6.0	9.4 ± 9.1
Urinary osmolality, mOsmol/kg	549 ± 10	552 ± 10	504 ± 32

ACE, angiotensin-converting enzyme; BP, blood pressure; CPB-time, cardio-pulmonary bypass time; cr, creatinine; cysC, cystatin C; eGFR, estimated glomerular filtration rate; EuroSCORE, European System for Cardiac Operative Risk Evaluation.

Data are reported as number of patients (%), mean ± SD or median (25th–75th percentile).

The number of missing values was for serum cystatin C (n = 43), smoking (n = 19), BMI (n = 14), APACHE IV score (n = 12), urinary creatinine (n = 4), urinary protein (n = 3), ACE-inhibitors (n = 3), statins (n = 3), antihypertensive medication (n = 3), EuroSCORE (n = 2), serum creatinine (n = 1). Other variables had no missing values.

^a^The APACHE IV score included information on age, temperature, mean arterial pressure, heart rate, respiratory rate, oxygenation, arterial pH, serum sodium and potassium, urine output, serum creatinine, liver function, hematocrit, white blood cell count, Glasgow Coma Scale, and presence of several chronic health conditions.

^b^The EuroSCORE consists of 17 items, including age, sex, chronic pulmonary disease, previous cardiac surgery, serum creatinine, left ventricular dysfunction, and whether a procedure was planned or emergency^22^.

^c^Diagnosis by a physician according to electronic medical records.

^d^Blood pressure-lowering drugs ATC codes C02, C03, C07, C08, and C09. Lipid-modifying drugs ATC code C10AA.

^e^Self-reported diagnosis by a physician, use of glucose-lowering drugs, or hyperglycemia.

^f^To convert the values for creatinine to mg/dL divide by 88.40.

### Profile of urinary biomarker levels before and after elective cardiac surgery

**Figure 1:**
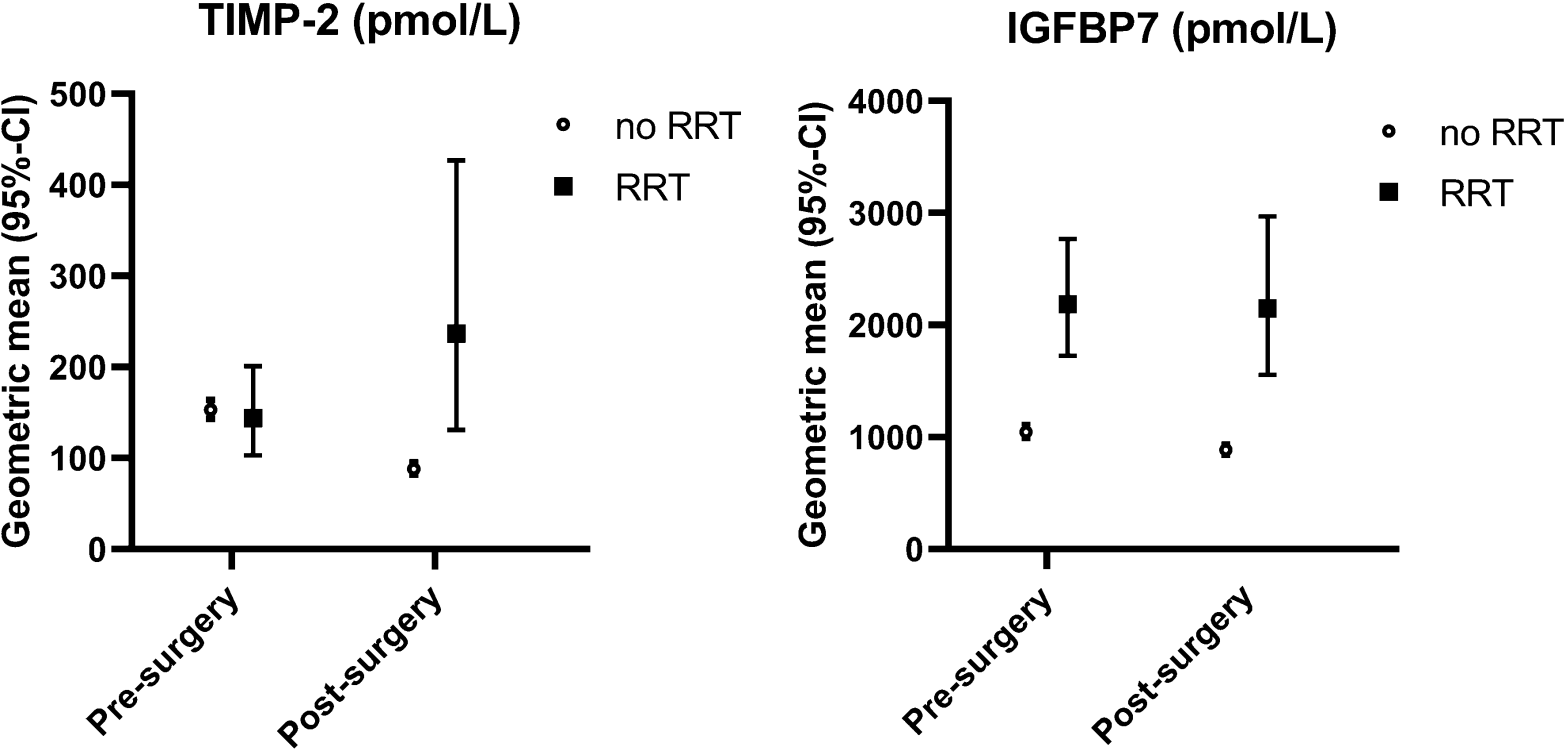
Urinary biomarker concentrations among cardiac patients, pre- and post-surgery according to need of renal replacement therapy. CI, confidence interval; IGFBP7, insulin-like growth factor-binding protein 7; RRT, renal replacement therapy; TIMP-2, tissue inhibitor of metalloprotease-2.

Pre-operative mean urinary TIMP-2 levels were comparable in patients who did or did not develop severe AKI necessitating RRT post-surgery However, post-operative TIMP-2 levels were 2.7-fold higher in patients who started RRT (Fig. 1, Supplementary Table S1). In contrast, IGFBP7 levels were consistently increased both pre- and post-surgery, in RRT patients compared with non-RRT patients. For the outcome duration of ICU stay ≥ 48 h and 30-day mortality, post-surgery TIMP-2 and IGFBP7 levels were about 1.5-fold and twofold higher, respectively (Supplementary Table S1).

### Discriminative value of urinary biomarkers

Pre-surgery TIMP-2 levels did not discriminate between patients who did or did not start RRT post-surgery (Fig. 1). Pre-surgery IGFBP7 reasonably discriminated need for RRT, with a C-statistic of 0.77 (95% CI 0.69; 0.85). However, baseline serum creatinine performed considerably better, with a C-statistic of 0.85 (0.75; 0.95). Combining serum creatinine with baseline IGFBP7 yielded a C-statistic of 0.92 (0.88; 0.96). Including the duration of surgery, as a proxy for procedural complications, also increased the C-statistic to 0.92 (0.88; 0.97). Despite the high C-statistic of a simple model consisting of baseline serum creatinine and duration of surgery, risk classification significantly improved upon addition of post-surgery IGFBP7 or TIMP-2 levels (Table 2). Odds ratios for each biomarker in relation to riskof RRT are presented in Supplementary Table S2. Calibration generally improved upon addition of each biomarker to the baseline pre- or post-surgery model, especially for IGFBP7 (Supplementary Fig. S2). The EuroSCORE model plus duration of surgery effectively predicted need for RRT, and improved upon adding either biomarker, especially IGFBP7 (Supplementary Table S3).

**Table 2:** Discrimination and reclassification performance with 95% CI of urinary biomarkers, change in biomarkers during surgery, and the biomarker product [TIMP-2] · [IGFBP7], regarding risk of renal replacement therapy.

	C-statistic^a^ 95% CI	C-statistic^a^ 95% CI	cf-NRI 95% CI	IDI 95% CI
Pre-surgery	Biomarker	+ serum creatinine		
	–	0.85 (0.75; 0.95)**	–	–
TIMP-2	0.45 (0.32; 0.58)	0.85 (0.74; 0.95)**	0.22 (− 0.23; 0.64)	− 0.002 (− 0.008; 0.003)
IGFBP7	0.77 (0.69; 0.85)*	0.92 (0.88; 0.96)**	0.80 (0.37; 1.23)*	0.029 (0.005; 0.052)*
[TIMP-2] · [IGFBP7]	0.58 (0.46; 0.70)	0.90 (0.86; 0.95)**	0.61 (0.18; 1.04)*	0.002 (− 0.011; 0.015)
Post-surgery	Biomarker	+ serum creatinine and surgery duration		
	–	0.92 (0.88; 0.97)**	–	–
TIMP-2	0.77 (0.66; 0.88)*	0.93 (0.90; 0.97)**	0.73 (0.29; 1.18)*	0.010 (− 0.029; 0.048)
IGFBP7	0.78 (0.69; 0.87)*	0.93 (0.87; 0.98)**	0.81 (0.37; 1.25)*	0.044 (− 0.001; 0.089)
TIMP-2 change^b^	0.76 (0.65; 0.87)*	0.92 (0.87; 0.98)**	0.61 (0.16; 1.05)*	0.011 (− 0.017; 0.038)
IGFBP7 change^b^	0.55 (0.45; 0.66)	0.92 (0.87; 0.97)**	0.30 (− 0.21; 0.81)	0.001 (− 0.004; 0.006)
[TIMP-2] · [IGFBP7]	0.80 (0.69; 0.91)*	0.92 (0.88; 0.97)**	0.82 (0.38; 1.26)**	0.033 (− 0.016; 0.082)

cf-NRI, category-free net reclassification improvement; CI, confidence interval; IDI, integrated discrimination improvement; IGFBP7, insulin-like growth factor-binding protein 7; TIMP-2, tissue inhibitor of metalloprotease-2.

*p < 0.05, **p < 0.001.

^a^Analyses were weighted towards the distribution of long *vs* short intensive care unit (ICU) stay of the original cohort.

^b^Change means the absolute difference between pre-surgery and post-surgery levels.

Change in IGFBP7 levels did not predict RRT (Table 2), while changes in TIMP-2 reasonably predicted RRT and improved risk classification of patients when added to a model containing baseline serum creatinine and duration of surgery. In general, C-statistics for the product of both biomarkers [TIMP-2] · [IGFBP7] were comparable to C-statistics for each biomarker individually (Table 2). Pre-surgery [TIMP-2] · [IGFBP7] poorly predicted RRT, with a C-statistic of 0.58 (95% CI 0.46; 0.70), while post-surgery the C-statistic was 0.80 (0.69; 0.91).

Pre-surgery biomarker levels did not predict 30-day mortality. In contrast, post-surgery levels significantly improved discrimination of a model consisting of serum creatinine or the EuroSCORE, and duration of surgery (Supplementary Tables S4, S5). The C-statistic for IGFBP7 alone was 0.80 (95% CI 0.73; 0.87) and was 0.87 (0.79; 0.94) for a model containing serum creatinine, duration of surgery, and post-surgery IGFBP7.

Results after multiple imputation were similar compared to the complete case analyses. Adjusting for urine osmolality did not change the results (Supplementary Table S6). A sensitivity analysis in which we classified the 3 patients who died within the first 7 days post-surgery into the group with RRT as outcome did not materially change our results (data not shown).

## Discussion

We showed in a cohort of elective cardiac surgery patients that urinary IGBP7 pre- and post-surgery or a change in TIMP-2 levels reasonably predicted the need for RRT with a C-statistic of about 0.80. Interestingly, a simple model consisting of baseline serum creatinine and duration of surgery had very good discriminative power with a C-statistic of 0.92, which was further improved to 0.93 upon addition of post-surgery TIMP-2 or IGFBP7. The product of [TIMP-2] · [IGFBP7] had a comparable performance as each biomarker individually. Post-surgery levels of TIMP-2 and IGFBP7 had reasonably to good discriminative value regarding 30-day mortality with C-statistics of 0.74 and 0.80, respectively.

To the best of our knowledge, no studies have investigated the potential role of the urinary biomarkers TIMP-2 or IGFBP7 for the prediction of RRT after elective cardiac surgery. Previous research included 50 to 100 patients, and studied the product of both biomarkers, [TIMP-2] · [IGFBP7] and the risk of AKI stage 2 or 3. However, these studies did not investigate each biomarker separately. Several studies found a good C-statistic for [TIMP-2] · [IGFBP7] 1 day post-surgery of > 0.80 regarding any stage of AKI^9–12^. Others reported an area under the curve (AUC) of about 0.5 immediately after surgery or 0.69 at 12 h post-surgery^13,14^. Bell et al. found that [TIMP-2] · [IGFBP7] did not predict AKI within 48 h after ICU admission in general ICU patients. Additionally, they showed that biomarker levels were significantly affected by comorbidities such as diabetes, challenging the robustness of these biomarkers^15^. A recent meta-analysis on the predictive value of urinary [TIMP-2] · [IGFBP7] concluded that it is an effective test for cardiac surgery-associated AKI, with a pooled C-statistic of 0.83^16^. However, all studies were pooled regardless of the timing of biomarker assessment. In contrast, in the present study we studied whether biomarker levels pre-surgery and directly post-surgery would be valuable predictors of AKI or RRT in an early stage. We found that both biomarkers directly post-surgery may be of value in predicting RRT. However, prediction of the need for RRT by a model containing only baseline serum creatinine and duration of surgery was already very high, leaving little room for improvement by additional biomarkers.

Data on the predictive value of both biomarkers with regard to mortality is scarce. Koyner et al. found that [TIMP-2] · [IGFBP7] did not improve the C-statistic of 0.70 of a clinical model predicting a composite outcome of death or dialysis within 9 months in critically ill patients. However, there was some improvement of both reclassification indices NRI and IDI^17^. Others showed in 98 critically ill patients that TIMP-2 predicted stage 3 AKI with a C-statistic of 0.80, and a C-statistic of 0.83 for 7-day mortality^18^. Importantly, critically ill patients are not comparable to elective cardiac surgery patients included in the present study. Critically ill patients consist of a heterogeneous population, admitted to the ICU for a variety of medical reasons, such as sepsis, coma, and respiratory insufficiency, and include emergency admissions.

We found twofold higher pre-surgery IGFBP7 levels in elective cardiac surgery patients who started RRT post-surgery versus patients not needing RRT. In contrast, pre-surgery TIMP-2 levels were comparable in patients who did or did not need RRT, but substantially increased post-surgery in patients who needed RRT. These results suggest that IGFBP7, which is mainly a proximal tubular marker, may be chronically elevated in part of the cardiac patients, while TIMP-2 increases only upon severe tubular damage. TIMP-2 levels especially increased during surgery in patients needing RRT. In line with these observations, in a cohort of kidney transplant patients, only TIMP-2 was a good predictor of delayed graft function. In contrast, IGFBP7 was elevated in most patients, which means discriminative value was poor^19^. Finally, we performed an additional analysis after adjusting urinary biomarker levels for urinary osmolality, since osmolality may influence biomarker levels^20^. We showed that results were comparable with or without adjustment for urinary osmolality.

This study had several limitations. First, because serum and urinary output measurements were recorded as part of routine care, the number of missing values for these variables was relatively high. Therefore, information on development of milder stages of AKI, e.g. stage 1 or 2, was not available. Additionally, sampling was terminated after patients left the ICU. We could therefore not continue monitoring biomarker levels after patients had left the ICU. Second, there were relatively few events of RRT or 30-day mortality, which prevented the incorporation of additional variables in our multivariable models. Nonetheless, discrimination was high using simple models, especially when predicting RRT.

The main strength of this study is the large homogenous sample of elective cardiac surgery patients. Second, we investigated different relevant endpoints, and additionally to standard methods, we used the newer indices for risk reclassification, NRI and IDI, as further measures of model improvement. Importantly, though both biomarkers measured post-surgery have good discrimination with regards to need for RRT, a biomarker with high discriminative value when measured pre-surgery would be much more clinically relevant. A predicted high risk for need for RRT or mortality post-surgery may aid in deciding which patients should be monitored more closely after surgery, but it has no implication on whether surgery should be performed in the first place.

In conclusion, we found that both TIMP-2 and IGFBP7 improved discrimination and risk classification of patients regarding RRT after elective cardiac surgery. Prediction of 30-day mortality was reasonable for both biomarkers. We found no evidence that the product [TIMP-2] · [IGFBP7] performed better than each biomarker alone. Discrimination regarding RRT after cardiac surgery was already very high using clinical variables such as baseline serum creatinine and duration of surgery. Nonetheless, in elective cardiac surgery patients, urinary TIMP-2 and IGFBP7 improved prediction of RRT post-surgery.

## Methods

### Participants

**Figure 2:**
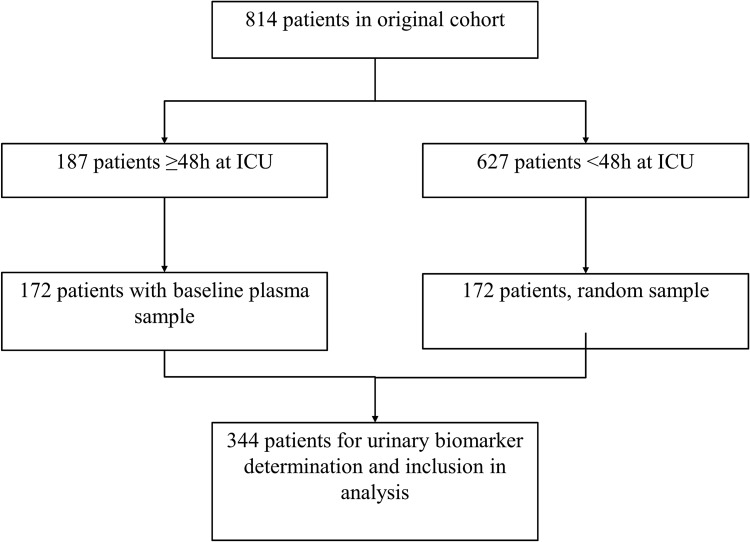
Flow chart of 344 patients available for analysis. ICU, intensive care unit.

This single-center observational study was performed at the Leiden University Medical Center, The Netherlands. The original cohort included 814 consecutive patients aged ≥ 18 years, undergoing elective cardiac surgery, between December 2006 and August 2010, as previously described in detail^21^. Exclusion criteria were pregnancy, active infection, and emergency surgery. After cardiac surgery, patients stayed in the ICU for post-operative care, according to usual clinical practice. After extubation and when hemodynamic and respiratory stable, patients were transferred to the thoracic surgery ward. For the present study, we selected all patients who stayed in the ICU for ≥ 48 h (n = 187) after cardiac surgery, and for whom a pre-operative plasma sample was available (92%; n = 172). We considered those who stayed < 48 h in the ICU as patients with a fast and relatively uncomplicated recovery from elective cardiac surgery (n = 627). For efficiency reasons, we randomly selected only 172 (28%) of these 627 patients for urinary biomarker assessment, taking into account calendar time. For each patient staying ≥ 48 h in the ICU, we selected a patient staying < 48 h in the ICU that was included in the study at about the same moment (Fig. 2). Patients randomly selected versus not selected patients were comparable with regard to baseline clinical characteristics (Supplementary Table S7). Analyses were weighted towards the distribution of time of stay in the ICU of the original cohort. Demographic and medical data, including the score of the European System for Cardiac Operative Risk Evaluation (EuroSCORE) model, were obtained from electronic medical records. The EuroSCORE model was developed in 1999 and is a validated prognostic scoring model to assess mortality risk after cardiac surgery^22^. The EuroSCORE reasonably predicted development of any stage of any AKI (C-statistic 0.70) and AKI stage 3 (C-statistic 0.78) in a cohort of 440 cardiac surgery patients^23^. The score consists of 17 items, including age, gender, chronic pulmonary disease, previous cardiac surgery, serum creatinine, left ventricular dysfunction, and whether a procedure was elective or an emergency. This study was conducted in accordance with the Helsinki protocol and standard of Good Clinical Practice, and was approved by the Medical Ethics Committee of the Leiden University Medical Center (CME-LUMC), Leiden, The Netherlands. All participants gave their written informed consent.

### Urinary measurements

Urine was sampled directly before surgery (baseline) and upon admission to the ICU. After sampling, urine was partitioned into 2 mL aliquots and stored at – 80 °C until analysis. Urine sampling was only done in the ICU and stopped when the patient was transferred to the ward.

Urinary TIMP-2 and IGFBP7 were quantified using sandwich enzyme-linked immunosorbent assays according to manufacturer’s instructions (ELISA, Cat. Nr. DTM200, R&D systems, Minneapolis, MN for TIMP-2, and Cat. Nr. EK0991, Boster Biological Technology, Pleasanton, CA for IGFBP7, respectively). Concentrations of urinary TIMP-2 and IGFBP7 after sample dilution were within the linear range. Low and high-level quality control (IQC) urine samples were prepared from pooled urine by spiking and analyzed in triplicate on each sample plate to assess the stability of the assay. Analysis for TIMP-2 was done with 3 different reagent lot numbers from April 2016 until October 2018. Mean TIMP-2 values (SD, %CV) of low and high IQC were 185 pmol/L (8 pmol/L, 4.4%, n = 45) and 257 pmol/L (22 pmol/L, 8.7%, n = 45), respectively. Analysis for IGFBP7 was done with 4 different reagent lot numbers, from April 2016 until October 2018. Mean IGFBP7 values (SD, %CV) of low and high IQC were 951 pmol/L (162 pmol/L, 17.0%, n = 70) and 2231 pmol/L (370 pmol/L, 16.6%, n = 70), respectively.

Osmolality was measured using Osmo-Station, ARKRAY Inc., Kyoto, Japan. We used normal, level 1, 376, and abnormal, level 2, 377 Lyphochek (BIO-RAD, Irvine, CA) quantitative urine controls for IQC, mean (SD, %CV) values were 320 mOsmol/kg (3 mOsmol/kg, 0.8%, n = 11) and 868 mOsmol/kg (7 mOsmol/kg, 0.8%, n = 10) for normal and abnormal quality control. Total protein (Cat. Nr 3333825190) and creatinine (Cat. Nr. 3263991190) were measured using a Cobas c502 analyzer (Roche Diagnostics, Mannheim, DE) according to the manufacturer’s instructions.

### Serum measurements

Serum samples were drawn immediately before cardiac surgery and upon arrival to the ICU. After mild centrifugation and partitioning the serum in 4 aliquots of 1 mL, serum was stored at – 80 °C until analysis. Serum cystatin C, 6600239190 and creatinine, 3263991190 were analyzed using a Cobas c502 analyzer, Roche Diagnostics, Mannheim, DE according to the manufacturer’s instructions. We used Cystatin C Control Set, 63729371190, for IQC when analyzing the serum cystatin C, and mean (SD, CV%) values were 1.10 mg/L (0.02 mg/L, 1.8%, n = 11), 1.57 mg/L (0.02 mg/L, 1.4%, n = 12) and 4.06 mg/L (0.05 mg/L, 1.3%, n = 11) for the CYSC2 Control 1, 2 and 3.

### Outcomes

The primary outcome was need for RRT within two weeks after surgery. As secondary outcome we investigated 30-day mortality.

### Statistical analyses

Baseline characteristics were presented as mean with standard deviation (SD), median (25th–75th percentile) or number (percentage), for all patients, and separately for patients who did and did not require RRT after cardiac surgery. Biomarker levels were log-transformed by the natural logarithm to normalize their distributions. For descriptive statistics, logarithmically transformed biomarker levels were back-transformed to the original scale to present geometric means and 95% confidence intervals (CIs). The proportion of missing values was 4.3% for baseline urinary TIMP-2 and IGFBP7, and 7.0% for urinary TIMP-2 and IGFBP7 levels post-surgery. The EuroSCORE could be determined in 99.4% of all patients and a baseline serum creatinine value was available for 99.7% of the entire cohort. There were no missing data on the outcomes RRT, long ICU stay, and 30-day mortality. In the main analyses we included complete cases only.

We used univariate logistic regression to assess the association of pre- and post-surgery biomarker levels, as well as the relative change between pre- and post-surgery levels, and occurrence of RRT and 30-day mortality. Discrimination of each biomarker was assessed by the C-statistic. We calculated C-statistics using weighted analyses, to adjust for the oversampling of patients with a short ICU stay, since unweighted analyses in case–control studies may lead to an underestimation of the C-statistic^24^.

We subsequently assessed whether IGFBP7 or TIMP-2 improved the performance of a pre-specified model containing a marker of kidney function, or a general ICU model to predict post-surgery mortality. Both models also included information on procedural complications. First, we assessed whether each biomarker improved a model containing baseline creatinine and duration of surgery. Pre-surgery models included only baseline serum creatinine, whereas post-surgery models also included duration of surgery. Duration of surgery was defined as the number of minutes between commencing and abolishing anaesthesia. Second, we assessed whether both biomarkers improved performance of the EuroSCORE model, regarding primary and secondary outcomes. As a more general model, we used the EuroSCORE model, which combines a variety of dichotomized parameters concerning health status into an additive score. We compared C-statistics of models with and without each biomarker. Since the C-statistic is a relatively insensitive measure for model improvement, we also assessed the category-free net reclassification improvement (cf-NRI) and integrated discrimination improvement (IDI)^25^. We have reported the cf-NRI, instead of the categorical NRI, because the categorical NRI required pre-defined risk categories and these are not available for TIMP-2 and IGFBP7. In such situations the cf-NRI is more appropriate^26^. Briefly, when calculating the cf-NRI, a new model is considered superior if a higher risk is assigned to an individual with the outcome, and a lower risk to an individual without the outcome, compared with the old model^27^. There are no official benchmarks for the cf-NRI, but values above 0.6 are suggested to indicate strong model improvement, values between 0.2 and 0.6 moderate, and less than 0.2 weak^28^. The IDI represents the difference in discrimination slopes between the old and new models^29^. The discrimination slope is the difference between the mean predicted risk in patients with versus without the outcome^29^. For the IDI, there are no cut-offs to determine the magnitude of model improvement. The NRI and IDI do not require weighting, provided that selected controls are a representative sample of the underlying cohort^24,30^. Calibration was assessed visually by calibration plots.

Finally, we assessed whether the absolute or relative difference between pre- and post-surgery biomarker levels predicted need for RRT. We also investigated the product of both biomarkers, [TIMP-2] · [IGFBP7], which has shown good predictive value in previous publications^8^.

### Sensitivity analyses

We repeated the analyses after multiple imputation, assuming data were missing at random. We used 10 imputations and included all relevant baseline variables and the outcome in the model. We derived standard errors of pooled estimates using Rubin’s rules^31^. We also repeated the analyses after adjusting urinary biomarkers for urinary creatinine and urine osmolality, to correct for physiological variation in urinary concentration. We have not performed competing risk analyses using death as competing risk for RRT, since only 3 patients died within the first 7 days post-surgery, and RRT was commenced within 7 days post-surgery for all patients. However, as sensitivity analysis we repeated the main analysis while including the 3 patients that died within 7 days post-surgery into the RRT group.

We considered two-sided *P* values < 0.05 statistically significant. All analyses were performed using STATA Statistical Software version 14 (Statacorp, Texas, USA) and SPSS 25.0 (IBM Corp., Armonk, NY, USA). We used the *idi* STATA package by Mark Lunt to calculate the NRI and IDI. We used the *pmcalplot* package to create calibration plots^32^.

## Supplementary Information

Supplementary Information.
