# Supplementary material for: The predictive value of TIMP-2 and IGFBP7 for kidney failure and 30-day mortality after elective cardiac surgery
Source: Sci Rep. 2021 Jan 13;11:1071. doi: 10.1038/s41598-020-80196-2 (PMC7806984; doi:10.1038/s41598-020-80196-2)
Supplement: Supplementary file 1 — Supplementary Information. [file 41598_2020_80196_MOESM1_ESM.docx]

**The predictive value of TIMP-2 and IGFBP7 for kidney failure and 30-day mortality after elective cardiac surgery**

**Running title: IGFBP7 and TIMP-2 for predicting acute kidney injury**

Kevin Esmeijer^1,2^, Abraham Schoe^3^, L. Renee Ruhaak^4^, Ellen K. Hoogeveen^1,2^, Darius Soonawala^1,5^, Fred P.H.T.M. Romijn^3^, Maryam R. Shirzada,^1^ Jaap T. van Dissel^3^, Christa M. Cobbaert^4^, Johan W. de Fijter^1^

1. Department of Nephrology, Leiden University Medical Center, Leiden, The Netherlands

2. Department of Clinical Epidemiology, Leiden University Medical Center, Leiden, The Netherlands

3. Department of Intensive Care, Leiden University Medical Center, Leiden, The Netherlands

4. Department of Clinical Chemistry and Laboratory Medicine, Leiden University Medical Center, Leiden, The Netherlands

5. Department of Internal Medicine, Haga Teaching Hospital, Den Haag, The Netherlands

*Corresponding author:*

Kevin Esmeijer, MD

Department of Nephrology, Leiden University Medical Center (building 1, C7-Q)

Albinusdreef 2, 2333 ZA Leiden, The Netherlands

Email: k.esmeijer@lumc.nl Tel: +31 71 526 56 20

**Supplementary Data**

**Supplementary Table S1:** **Urinary biomarker level pre- and post-surgery in cardiac surgery patients according to need for RRT and 30-day mortality.**

|  | TIMP-2 (pmol/L)  Geometric mean (95% CI) | | IGFBP7 (pmol/L)  Geometric mean (95% CI) | |
| --- | --- | --- | --- | --- |
|  | Pre-surgery | Post-surgery | Pre-surgery | Post-surgery |
| Renal replacement therapy |  |  |  |  |
| No | 153 (143; 164) | 88 (82; 95) | 1046 (981; 1116) | 884 (830; 941) |
| Yes (n=22) | 144 (103; 201) | 237 (131; 427) | 2186 (1726; 2769) | 2147 (1554; 2967) |
| Ratio | 0.9 (0.7; 1.3) | 2.7 (1.7; 4.3) | 2.1 (1.6; 2.7) | 2.4 (1.7; 3.1) |
| p for ratio ^a^ | 0.7 | <0.001 | <0.001 | <0.001 |
|  |  |  |  |  |
| 30-day mortality |  |  |  |  |
| No | 154 (144 (165) | 89 (82; 96) | 1034 (998; 1134) | 889 (475; 1663) |
| Yes (n=18) | 117 (75; 185) | 179 (112; 288) | 1300 (864; 1956) | 1821 (1262; 2627) |
| Ratio | 0.8 (0.5; 1.1) | 2.0 (1.2; 3.5) | 1.2 (0.8; 1.8) | 2.0 (1.3; 3.2) |
| p for ratio ^a^ | 0.2 | 0.02 | 0.3 | 0.001 |

CI, confidence interval; ICU, intensive care unit; IGFBP7, insulin-like growth factor-binding protein 7; RRT, renal replacement therapy; TIMP-2, tissue inhibitor of metalloprotease-2.

^a^ Ratio means relative difference in biomarker levels between both groups.

**Table S2: Odds ratios for the outcome RRT, for each biomarker individually and in a multivariable model, resulting from logistic regression analyses.**

| Model | OR (95%-CI) for RRT |
| --- | --- |
| Pre-surgery |  |
| TIMP-2^a^ | 0.95 (0.68; 1.31) |
| TIMP-2^a^ adjusted for serum creatinine | 0.98 (0.56; 1.70) |
|  |  |
| IGFBP7^a^ | 2.51 (1.70; 3.71) |
| IGFBP7^a^ adjusted for serum creatinine | 2.87 (1.76; 4.70) |
|  |  |
| Post-surgery |  |
| TIMP-2^a^ | 2.99 (1.46; 6.11) |
| TIMP-2^a^ adjusted for serum creatinine and surgery duration | 1.90 (0.71; 5.12) |
|  |  |
| IGFBP7^a^ | 3.68 (1.93; 6.99) |
| IGFBP7^a^ adjusted for serum creatinine and surgery duration | 3.75 (1.47; 9.52) |

CI, confidence interval; IGFBP7, insulin-like growth factor-binding protein 7; OR, odds ratio; RRT, renal replacement therapy; TIMP-2, tissue inhibitor of metalloprotease-2.

^a^ Odds ratios are presented per 1 unit increment log-transformed TIMP-2 or IGFBP7.

**Supplementary Table S3: Discrimination and reclassification performance of urinary biomarkers regarding risk of renal replacement therapy, and improvement of the EuroSCORE model.**

|  | C-statistic ^a^  95% CI | cf-NRI  95% CI | IDI  95% CI |
| --- | --- | --- | --- |
| Pre-surgery |  |  |  |
| TIMP-2 | 0.45 (0.32 (0.58) | - | - |
| IGFBP7 | 0.77 (0.69; 0.85) | - | - |
| EuroSCORE | 0.65 (0.55; 0.75) | - | - |
| EuroSCORE + TIMP-2 | 0.65 (0.55; 0.76) | 0.32 (-0.11; 0.75) | 0.0004 (-0.0008; 0.0016) |
| EuroSCORE + IGFBP7 | 0.80 (0.73; 0.88)^b^ | 0.71 (0.28; 1.14)* | 0.018 (0.002; 0.033)* |
|  |  |  |  |
| Post-surgery |  |  |  |
| TIMP-2 | 0.77 (0.66; 0.88) | - | - |
| IGFBP7 | 0.78 (0.68; 0.89) | - | - |
| EuroSCORE + surgery duration | 0.79 (0.71; 0.88) | - | - |
| EuroSCORE + surgery duration + TIMP-2 | 0.81 (0.69; 0.92) | 0.58 (0.12; 1.03)* | 0.089 (0.022; 0.156)* |
| EuroSCORE + surgery duration + IGFBP7 | 0.84 (0.74; 0.94) | 1.02 (0.57; 1.47)** | 0.098 (0.051; 0.144)* |

cf-NRI, category-free net reclassification improvement; EuroSCORE, European System for Cardiac Operative Risk Evaluation; IDI, integrated discrimination improvement; IGFBP7, insulin-like growth factor-binding protein 7; TIMP-2, tissue inhibitor of metalloprotease-2

* p <0.05, ** p <0.001

^a^ Analyses were weighted towards the distribution of long *vs* short ICU stay of the original cohort.

^b^ Statistically significant improvement of C-statistic, compared to a model containing the EuroSCORE alone.

**Supplementary Table S4: Discrimination and reclassification of urinary biomarkers regarding the outcome 30-day mortality, and improvement of a renal model.**

|  | C-statistic ^a^ | cf-NRI (95% CI) | IDI |
| --- | --- | --- | --- |
| Pre-surgery |  |  |  |
| TIMP-2 | 0.41 (0.26; 0.56) | - | - |
| IGFBP7 | 0.58 (0.44; 0.72) | - | - |
| Serum creatinine^b^ | 0.63 (0.50; 0.75) | - | - |
| Serum creatinine^b^ + TIMP-2 | 0.66 (0.53; 0.78) | -0.05 (-0.52; 0.42) | -0.0003 (-0.001; 0.001) |
| Serum creatinine^b^ + IGFBP7 | 0.63 (0.48; 0.78) | 0.37 (-0.10; 0.84) | 0.005 (-0.003; 0.013) |
|  |  |  |  |
| Post-surgery |  |  |  |
| TIMP-2 | 0.74 (0.63; 0.85) | - | - |
| IGFBP7 | 0.80 (0.73; 0.87) | - | - |
| Serum creatinine^b^ + surgery duration | 0.75 (0.64; 0.86) | - | - |
| Serum creatinine^b^ + surgery duration + TIMP-2 | 0.80 (0.70; 0.91) | 0.43 (-0.12; 0.98) | 0.004 (-0.008; 0.016) |
| Serum creatinine^b^ + surgery duration + IGFBP7 | 0.87 (0.79; 0.94) ^c^ | 0.63 (0.08; 1.18)* | 0.012 (-0.015; 0.039) |

cf-NRI, category-free net reclassification improvement; CI, confidence interval; IDI, integrated discrimination improvement; IGFBP7, insulin-like growth factor-binding protein 7; TIMP-2, tissue inhibitor of metalloprotease-2

* p <0.05

^a^ Analyses were weighted towards the distribution of long *vs* short ICU stay of the original cohort.

^b^ Serum creatinine at baseline, before surgery.

^c^ Statistically significant improvement of C-statistic, compared to a model including serum creatinine + surgery duration.

**Supplementary Table S5: Discrimination and reclassification of urinary biomarkers regarding the outcome 30-day mortality, and improvement of the EuroSCORE model.**

|  | C-statistic ^a^ | cf-NRI (95% CI) | IDI |
| --- | --- | --- | --- |
| Pre-surgery |  |  |  |
| TIMP-2 | 0.41 (0.26; 0.56) | - | - |
| IGFBP7 | 0.58 (0.44; 0.72) | - | - |
| EuroSCORE | 0.68 (0.58; 0.77) | - | - |
| EuroSCORE + TIMP-2 | 0.70 (0.59; 0.82) | 0.22 (-0.25; 0.69) | 0.0001 (-0.001; 0.001) |
| EuroSCORE + IGFBP7 | 0.69 (0.61; 0.78) | 0.17 (-0.30; 0.64) | 0.001 (-0.006; 0.008) |
|  |  |  |  |
| Post-surgery |  |  |  |
| TIMP-2 | 0.74 (0.63; 0.85) | - | - |
| IGFBP7 | 0.80 (0.73; 0.87) | - | - |
| EuroSCORE + surgery duration | 0.77 (0.70; 0.84) | - | - |
| EuroSCORE + surgery duration + TIMP-2 | 0.80 (0.69; 0.91) | 0.76 (0.21;1.31)* | 0.018 (0.003; 0.033)* |
| EuroSCORE + surgery duration + IGFBP7 | 0.85 (0.79; 0.92) ^b^ | 0.81 (0.26; 1.36)* | 0.019 (0.004; 0.034)* |

cf-NRI, category-free net reclassification improvement; CI, confidence interval; EuroSCORE, European System for Cardiac Operative Risk Evaluation; IDI, integrated discrimination improvement; IGFBP7, insulin-like growth factor-binding protein 7; TIMP-2, tissue inhibitor of metalloprotease-2

* p <0.05

^a^ Analyses were weighted towards the distribution of long *vs* short intensive care unit (ICU) stay of the original cohort.

^b^ Statistically significant improvement of C-statistic, compared to a model including the EuroSCORE + surgery duration.

**Supplementary Table S6: Discrimination and reclassification of osmolality-adjusted urinary biomarkers regarding the outcome renal replacement therapy (RRT).**

|  | C-statistic ^a^ | cf-NRI | IDI |
| --- | --- | --- | --- |
| Pre-surgery |  |  |  |
| TIMP-2 | 0.60 (0.48; 0.72) | - | - |
| IGFBP7 | 0.67 (0.56; 0.78) | - | - |
| Serum creatinine | 0.85 (0.74; 0.96) | - | - |
| Serum creatinine + TIMP-2 | 0.84 (0.73; 0.96) | -0.24 (-0.69; 0.21) | -0.000 (-0.0004; 0.0004) |
| Serum creatinine + IGFBP7 | 0.85 (0.74; 0.96) | 0.22 (-0.23; 0.67) | 0.003 (-0.001; 0.007) |
|  |  |  |  |
| Post-surgery |  |  |  |
| TIMP-2 | 0.75 (0.63; 0.87) | - | - |
| IGFBP7 | 0.75 (0.64; 0.86) | - | - |
| Serum creatinine + surgery duration | 0.92 (0.88; 0.97) | - | - |
| Serum creatinine + surgery duration + TIMP-2 | 0.93 (0.89; 0.96) | 0.48 (0.03; 0.93)* | -0.0004 (-0.014; 0.013) |
| Serum creatinine + surgery duration + IGFBP7 | 0.92 (0.87; 0.96) | 0.41 (-0.04; 0.86) | 0.003 (-0.004; 0.010) |

cf-NRI, category-free net reclassification improvement; IDI, integrated discrimination improvement; IGFBP7, insulin-like growth factor-binding protein 7; TIMP-2, tissue inhibitor of metalloprotease-2

* p <0.05

^a^ Analyses were weighted towards the distribution of long *vs* short intensive care unit (ICU) stay of the original cohort.

**Supplementary Table S7: Baseline characteristics for patients with a short ICU stay <48 hours, separately for patients randomly selected into the analyses, and patients not selected.**

|  | Selected patients (n=172) | Not selected (n=455) | p-value for difference |
| --- | --- | --- | --- |
| Age, years | 66 (11) | 65 (12) | 0.3 |
| Duration of surgery, min | 276 (106) | 279 (84) | 0.7 |
| EuroSCORE | 5.3 (2.7) | 5.3 (2.9) | 0.9 |
| Male sex, no (%) | 109 (63) | 301 (69) | 0.2 |
| Heart failure, no (%) | 21 (12) | 88 (20) | 0.02 |
| Diabetes, no (%) | 35 (20) | 71 (16) | 0.2 |
| Smoking, no (%) | 34 (20) | 134 (30) | 0.02 |
| Hypertension, no (%) | 80 (47) | 196 (43) | 0.4 |
| APACHE IV score | 47 (13) | 46 (14) | 0.5 |

Values represent mean (SD) for continuous variables, and number (%) for categorical variables. Continuous variables were compared using an independent samples T-test, categorical variables were compared using a Chi-squared test.


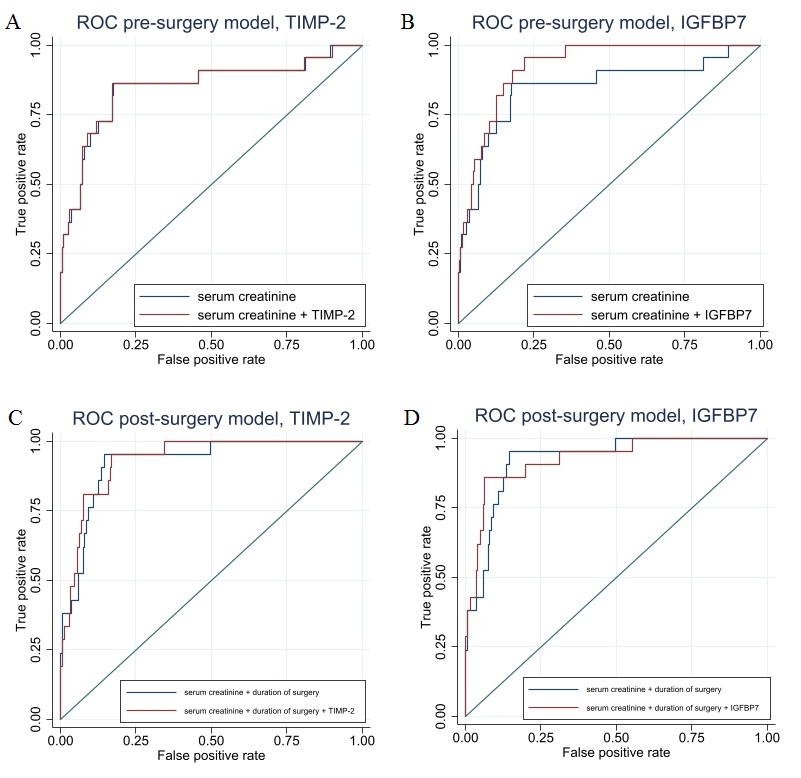


**Figure S1: ROC curves of pre- and post-surgery models with and without addition of biomarkers TIMP-2 and IGFBP7, for the outcome RRT.** Figure A: pre-surgery model for serum creatinine alone (C-statistic 0.85) and with TIMP-2 (C-statistic 0.85). Figure B: pre-surgery model for serum creatinine alone and with IGFBP7 (C-statistic 0.92). Figure C: post-surgery model for serum creatinine plus duration of surgery alone (C-statistic 0.92) and with TIMP-2 (C-statistic 0.93). Figure D: post-surgery model for serum creatinine and duration of surgery alone and with IGFBP7 (C-statistic 0.93). 2 and IGFBP7, respectively.


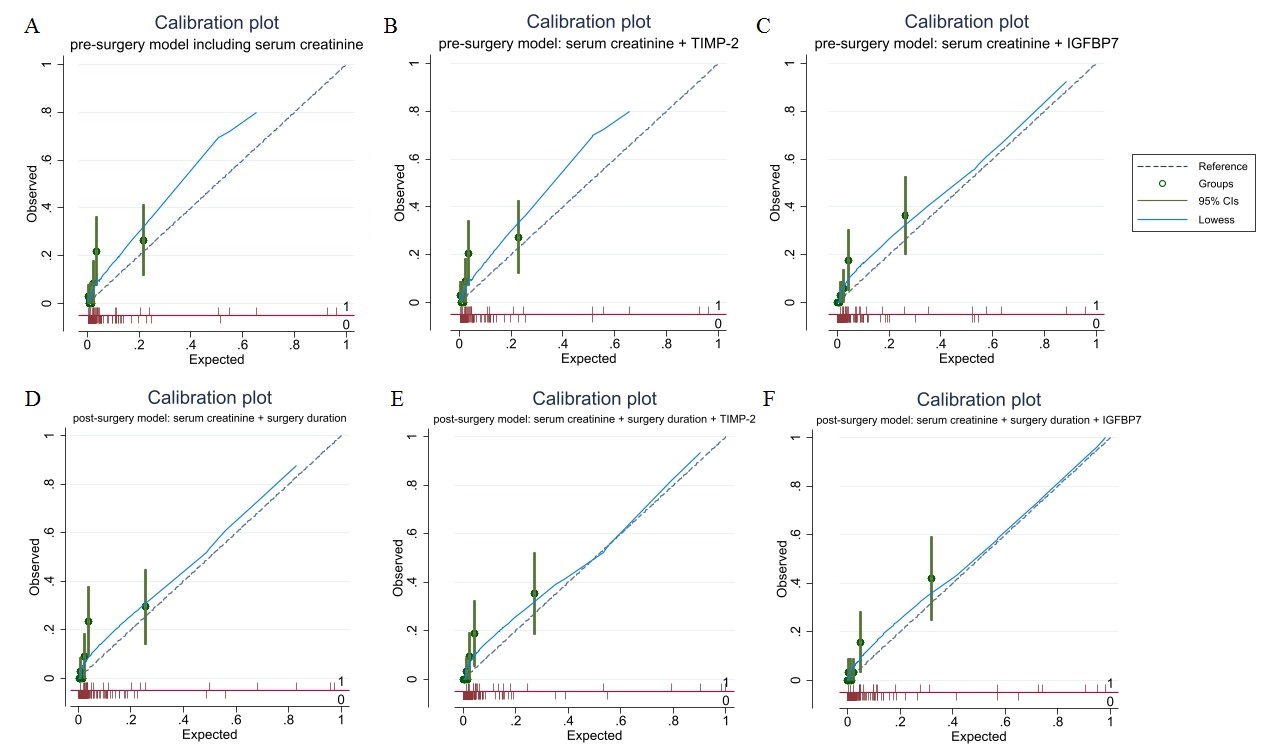


**Figure S2: Calibration plots for pre- and post-surgery models with and without addition of biomarkers, for the outcome RRT.** Panel A, B and C represent pre-surgery models without biomarkers, with TIMP-2, and with IGFBP7, respectively. Panel D, E and F represent post-surgery models without biomarkers, with TIMP-2, and with IGFBP7, respectively. The spike plots at the bottom of each graph show the number of events (1) and non-events (0) over the range of expected probabilities. Groups represent deciles of predicted risk.
